# Intravitreal Ampicillin Sodium for Antibiotic-Resistant Endophthalmitis: *Streptococcus uberis* First Human Intraocular Infection Report

**DOI:** 10.1155/2010/169739

**Published:** 2010-07-14

**Authors:** Raul Velez-Montoya, Dulce Rascón-Vargas, William F. Mieler, Jans Fromow-Guerra, Virgilio Morales-Cantón

**Affiliations:** ^1^Retina Department, Associacition to prevent blindness in México IAP, México City, Mexico; ^2^Department of Ophthalmology and Visual Science, University of Illinois, Chicago, IL 60607, USA

## Abstract

*Purpose*. To describe the clinical characteristics, diagnosis, and treatment with intravitreal ampicillin sodium of a postoperative endophthalmitis case due to *Streptococcus uberis*; an environmental pathogen commonly seen in mastitis cases of lactating cows. *Methods*. Case Report. A 52-year-old, Hispanic diabetic patient who suddenly developed severe pain and severe loss of vision, following vitrectomy. *Results*. The patient was diagnosed with postoperative endophthalmitis secondary to a highly resistant strain of *Streptococcus uberis* that did not respond to intravitreal antibiotics. He was treated with an air-fluid interchange, anterior chamber washout, intravitreal ampicillin sodium (5 mg/0.1 mL), and silicon oil tamponade (5000 ck). The eye was anatomically stabilized, though there was no functional recovery. *Conclusion*. *Streptococcus uberis* is an uncommon pathogen to the human eye, which has unique features that help the strain in developing resistance to antibiotics. While treatment with intravitreal ampicillin is feasible, there are still concerns about its possible toxicity.

## 1. Introduction

Endophthalmitis is a rare postoperative complication which is potentially devastating to visual function and the structural integrity of the eye [1]. In the postoperative setting, infection generally occurs secondary to contamination with normal periocular flora. Occasionally, it develops from sources which are difficult to identify. Once detected postoperatively, the condition is treated with intravitreal antibiotics and vitrectomy and/or tap as per the recommendations of the Endophthalmitis Vitrectomy Study (EVS) [2].

In recent years, there has been an increase of the number of antibiotic-resistant bacterial strains and new strains which are normally not part of the traditional etiological spectrum of postoperative infection [3, 4]. The following case report has the objective of describing the diagnosis, treatment, and unfavorable evolution of one case of postoperative endophthalmitis, secondary to *Streptococcus uberis. *This environmental pathogen is commonly responsible for a high proportion of cases of clinical (and subclinical) mastitis in lactating cows [5]. The organism is highly resistant to the majority of the latest generation antibiotics which are commonly employed in the treatment of endophthalmitis. Precisely how this patient became exposed to this pathogen remains unclear.

## 2. Case Report

A 52-year-old, Hispanic male presented to the retina department of our hospital complaining of a three-month history of progressive visual loss in his left eye. His past medical history was remarkable for Diabetes Mellitus (18 years) with poor metabolic control (last glucose level was 167 mg/dL, with a HbA1C of 14.7%), high blood pressure, chronic renal failure (treated with peritoneal dialysis), and diabetic ischemic foot problems (previous amputation of three toes). The patient also had history of previous abdominal surgeries (23 years ago). 

As for the ophthalmologic background, the patient had a previous diagnosis of proliferative diabetic retinopathy, which had been treated previously with bilateral panretinal photocoagulation, and vitrectomy OD along with chronic open angle glaucoma OU. 

The best corrected visual acuity was 20/40 in OD and counting fingers at 30 cm in OS, and the anterior chamber examination was unremarkable. Ocular motility and pupillary responses were normal. The lens in the left eye was cataractous (C2N3P2, according to LOCS III classification), and intraocular pressure was 16 mmHg OU. Fundus examination revealed a dense vitreous hemorrhage in the left eye. Ultrasound examination of the left eye confirmed the presence of low reflective mobile vitreous opacities, consistent with vitreous hemorrhage, despite not show, evidence of traction retinal detachment. 

Based on the existing evidence, we decided to offer the patient phacoemulsification surgery combined with a 23 GA vitrectomy. The surgery was performed without complications shortly after the initial examination, leaving balanced saline solution in the vitreous cavity at the end of the procedure. Although the vitrectomy ports were self-sealing, we decided to place a suture (8-0 Vicryl, Ethicon, San Angelo TX, USA) in all of them. We also placed a suture in the phacoemulsification incision (10-0 Nylon, Ethicon, San Angelo TX, USA). 

**Figure 1:**
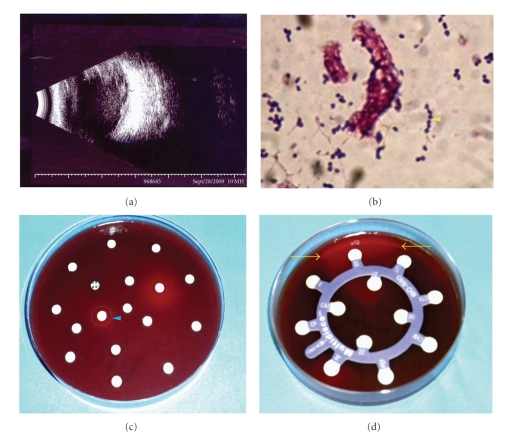
(a): Patient's B-mode ultrasonography, which shows areas of increased echogenicity in the vitreous cavity. (b) Vitreous cavity sample gram stain that shows gran-positive cocci. ((c) and (d)) Antibiotic sensitivity tests. Blue arrow head: mild bacterial growth inhibition secondary to moxifloxacin disc. Yellow arrows: complete bacterial growth inhibition due to ampicillin sodium disc.

Twenty-four hours after surgery, the patient complained of severe ocular pain, along with significant reduction of visual acuity (Hand Movements) and tearing. On ocular examination, we found severe conjunctival hyperemia, ciliary injection, clear cornea, hypopyon in the anterior chamber (1.2 mm), and intraocular pressure of 30 mmHg. The posterior pole was not visible. The ultrasound examination revealed images of increased echogenicity which correspond to cellularity in vitreous cavity, pseudomembranes formation, and choroidal thickening (Figure 1(a)). The diagnosis of postoperative endophthalmitis was evident, and we proceeded to immediately obtain aqueous and vitreous cavity samples for staining, cultures, and sensitivity tests. Intravitreal Ceftazidime (2.25 mg/0.1 mL), Vancomycin (1 mg/0.1 mL), and Dexamethasone (0.4 mg/0.1 mL) were injected. The patient was admitted to the hospital, and treatment was started with topical moxifloxacin every hour (Vigamox, Alcon Lab, Dallas Fort worth, TX) and oral moxifloxacin (400 mg). The following day, the visual acuity decreased to no light perception and severe pain and hypopyon continued. At the same day, the microbiology department reported the presence of gram-positive cocci in the vitreous cavity sample (which was classified as *Streptococcus uberis* two days later (Figure 1(b)). The sensitivity test documented resistance to cephalothin, cefotaxime, ceftazidime, cefuroxime, dicloxacillin, vancomycin, azithromycin, clarithromycin, erythromycin, amikacin, gentamicin, netilmicin, tobramycin, clindamycin polymyxin, ciprofloxacin, gatifloxacin, moxifloxacin, ofloxacin, perfloxacin, and tetracycline (Figures 1(c) and 1(d)). The only known sensitivity was to ampicillin sodium. 

Due to the lability of the patient and the possibility of systemic dissemination of the bacteria, we offered to the patient an air-fluid exchange, silicone oil as tamponade, anterior chamber washout, and intraocular lens removal after the failure of the first intravitreal antibiotics. However, the patient refused to sign the informed consent form for the second surgery, delaying treatment for three days. After knowing the specific sensitivity of the microorganism, we added an intravitreal injection of ampicillin sodium 5 mg/0.1 mL to the original plan. Finally, after extensive and exhaustive explanation, the patient agreed to the procedure. 

**Figure 2:**
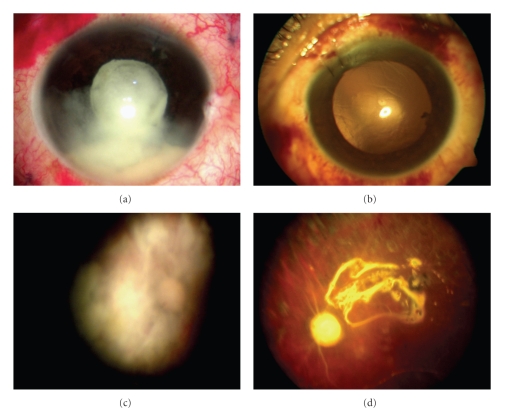
Patient's clinical evolution: ((a) and (c)) previous air-fluid interchange and intravitreal ampicillin sodium; the anterior segment biomicroscopy showed severe conjunctival hyperemia, severe cellularity, and Hypopyon with a fibrin clot on the surface of the intraocular lens. The details of the retina cannot be observed due to significant posterior opacities. However, the optic nerve seems to be extremely pale with severe attenuation of the blood vessels. ((b) and (d)) Four weeks after second surgery; anterior segment biomicroscopy showed an improvement in conjunctival hyperemia (mild), with disappearance of Hypopyon and fibrin cloth, and the retina showed extensive areas of photocoagulation (first surgery), severe pallor of the optic nerve, bloodless vessels, and vitreous cavity filled with silicon oil.

The next day, the patient reported decreased pain, and on examination the vision remained no light perception, though there was no evidence of hypopyon and only mild conjunctival hyperemia. The patient remained hospitalized for the next three days, and during that time ampicillin sodium was administered intravenously, at adjusted doses of 1000 mg bid according to creatinine clearance. After discharge, the patient continued treatment with maintenance doses of intramuscular ampicillin sodium for two weeks. The patient continued to improve. Four weeks later, the integrity of the eye was preserved but the vision remained no light perception (Figure 2). 

## 3. Discussion

Despite the advances in surgical techniques and the technology available to perform ocular surgery, the incidence of postoperative endophthalmitis in the last 10 years appears to be increasing [6, 7]. What possibly play a role in this development has been the indiscriminate and inappropriate dosing of broad-spectrum antibiotics by doctors and the inadequate compliance to full treatment duration by the patients. This has led to the emergence of new resistant strains to the latest generations of drugs [3, 4, 6]. Evidence of this has been seen in the results published by the Ocular Tracking Resistance in the U.S. Today (TRUST) program, which reported an increase of 12.1% of methicillin-resistant *Staphylococcus aureus* (MRSA) strains, with more than 80% of MRSA being resistant to fluoroquinolones. However, despite the considerable increase of this numbers, it is also important to note that the study has the limitation that they based the bacterial susceptibility to antibiotics on systemic drug-exposure breakpoints and not in local concentration (as in an intravitreal injection) [6]. 

*Streptococcus uberis* is an environmental pathogen which is typically responsible for mastitis cases in lactating cows. It is also the predominant organism isolated from mammary glands during the nonlactating period in cows. Although *β*-lactams are the treatment of choice, the bacteria possess unique mechanism to generate resistance to antibiotics like the *mph*(B) gene for resistance to macrolide and SOS response-like DNA repair mechanism which may induce SOS-driven adaptive mutations [5, 8]. The uncommon strong resistance to antibiotics found in the strain cultured from the patient's vitreous samples could be the result of all these conditions. The reason and circumstances by which this microorganism was able to reach the eye remains hidden to all of us. 

Since there was no improvement clinically of our patient after the first intravitreal injection, and the isolated organism was resistant to practically all the intravitreal antibiotics that are commonly employed, we decided to use the only antibiotic to which the organism appeared to be sensitive. Our use of 5 mg/0.1 mL of intravitreal ampicillin sodium was based on two previous reports in which the intravitreal administration proved to be safe. Those reports were based on unpublished data from G. A. Peyman in which he established that the ampicillin sodium could be safely administered intraocularly up to a dose of 10 mg/0.1 mL. However, although the results were published in his book, the original study was never published [9, 10].

The fact that almost all the traditional pathogens responsible for endophthalmitis cases are beta-lactamase producing strains limits the use of this antibiotic as part of the first choice drugs for the treatment of postoperative endophthalmitis. The possibility of toxicity-induced damage due to ampicillin sodium is also a factor to be considered, although this patient's vision already showed no light perception prior to administration of the intravitreal ampicillin. In this case, the eye was anatomically salvaged with this treatment regimen, although without visual recovery.
